# Utilization of growth monitoring and promotion services and associated factors among under two years of age children in Southern Ethiopia

**DOI:** 10.1371/journal.pone.0177502

**Published:** 2017-05-16

**Authors:** Fentaw Wassie Feleke, Anchamo Anato Adole, Afework Mulugeta Bezabih

**Affiliations:** 1 Mareka District Health Office, Waka, Ethiopia; 2 College of Agriculture, Hawasssa University, Hawassa, Ethiopia; 3 College of Medicine and Health sciences, Mekelle University, Mekele, Ethiopia; Centre Hospitalier Universitaire Vaudois, FRANCE

## Abstract

Growth monitoring and promotion (GMP) is a prevention activity comprised of growth monitoring (GM) linked with promotion that serves as the core activity in an integrated child health and nutrition program. However, different methods of institutional studies have shown that utilization of GM services has remained to be inadequate. There is scarcity of studies conducted about GMP in quantitative method. Therefore, this study was conducted to address the proportion of GMP services and associated factors among children under two years of age in rural communities of Mareka district, Southern Ethiopia. Community based cross-sectional survey was conducted from August to September 2015. Single population proportion formula was used to determine the sample size with multi stage sampling technique. A total of 819 children under two years of age were included. Pretest was done on 10% of the total sample size. Data were analyzed using SPSS version 20.0 software. Bivariate and multivariate logistic regressions used to analyze data. The response rate was 95%. Utilization of GMP services was 16.9%. Institutional delivery AOR (95% CI): 3.01(1.65–5.50), index child age 12–17 months AOR (95% CI): 4.03(2.16–7.51) and 18–23 months AOR (95% CI): 3.08(1.70–5.57), family size 4–5 AOR(95% CI): 0.14(0.06–0.33), family size >5 AOR(95% CI): 0.34(0.14–0.82), regular GMP attendance AOR (95% CI): 4.37(2.45–7.80), medium wealth index AOR(95% CI): 3.14(1.51–6.52) and high wealth index AOR(95% CI): 3.24(1.59–6.62) were factors associated with utilization of GMP services. Utilization of GMP services was low. Thus, efforts should be made to improve utilization of GMP services through promotion of institutional delivery, different family planning methods, and regular GMP attendance.

## Introduction

Malnutrition remains the world’s most serious health problem and the single biggest contributor to child mortality [1]. Directly or indirectly malnutrition is responsible for over half of all childhood deaths[2]. Child malnutrition is a serious public health problem in Ethiopia. According to central statistical agency in Ethiopia 40% of children under five were stunted, 25% were underweight and 9% were wasted. Similarly in Southern Ethiopia 44.3% of children were stunted, 26.3% were underweight and 6.8% were wasted [3].

The Ethiopian government has been implementing GMP services at community level through health extension programs in order to improve child nutritional status. GMP is a prevention activity comprised of GM linked with promotion that increases awareness about child growth; improves caring practices; increases demand for other services, as needed; and serves as the core activity in an integrated child health and nutrition programme[4].

Even though there are many efforts including GMP services at grass root level to reduce child malnutrition in Ethiopia, prevalence of malnutrition is still high among under five children. There are no studies conducted in Ethiopia on utilization of GMP services and associated factors among under two years of age children. To the best of the investigators’ knowledge, there were no previous quantitative studies characterized factors associated with utilization of GMP services in Ethiopia except one qualitative study from Northern Ethiopia [5]. Therefore this study was conducted to assess utilization GMP services and associated factors among under two years of age children in rural area of Mareka district, Southern Ethiopia.

## Materials and methods

### Study design, area and period

A community based cross-sectional study design was used in rural area of Mareka district, Southern Ethiopia from August to September 2015.

### Source, study population and study unit

Source populations were all mother-child pairs with 0–23 months in study area. Study populations were all mother-child pairs with 0–23 months from randomly selected kebeles. Mothers who gave information about their children 0–23 months were the study unit.

### Sample size determination and sampling procedure technique

Sample size was calculated using single population proportion formula with the assumptions of: 95% confidence level, proportion of GMP services utilization (59%) for children 0–23 months old, margin of error (5%), design effect (2) and contingency for non-response (10%); n = $\frac{2\;\text{X}[1.96^{2}\text{X}\;0.59(1\text{-}0.59)]}{0.05^{2}}$ = 743.426432 ~ 744, adding 10% of non-response rate ~ 75, final sample size n = 819. Multi stage sampling technique was used with purposive selection of study district. When study participants were absent during data collection, the next closest household with eligible criteria was chosen. The youngest child in the restricted age group was taken as an index child. When twin children were found within one household, data were collected from one of them by lottery method.

### Data collection procedures

Data were collected using face-to-face interviewer and pretested structured questionnaire. Sociodemographic, economic, maternal, and child characteristics questionnaires were adopted from EDHS 2011. WHO 2006 GMP chart was used.

### Operational definition

#### Utilization of GMP services

Participation of a child for GMP services at least once for 0 month, at least two times for 1–3 months, at least five times for 4–11 months and at least four times per year for 12–23 months [6].

### Data processing and analysis

Data were entered using Epi version 3.1.2.7 and exported to SPSS Version 20.0 software for analysis. Cleaning was conducted 100%. Different proportions were calculated and presented as descriptive statistics. Bivariate and multivariate logistic regressions were done to identify the predictors. According to Hosmer and Lemeshow theory of ten cases per independent variables about eleven independent variables that were remained statistically significant in bivariate analysis with (p<0.001) except wealth index entered to multivariable logistic regression model. The model fitness was checked by using Hosmer-Lemeshow goodness of fit test with (p = 0.43). Multi-collinearity diagnosis was ruled-out with all the variance inflation factors being less than two. Finally, statistical association was declared at 95% confidence interval and adjusted odds ratio [7]. Wealth index was assessed using household assets via principal component analysis adopted from [3].

### Ethical considerations

Ethical clearance was obtained from the Institutional Review Board of Hawassa University. Written permission was obtained from Zonal and Woreda administration. Written consent was obtained from the mothers/caregivers after informing all the purpose, benefits, and risks of the study. These consent procedures were approved by Hawassa University Institutional Review Board ethical committee.

## Results

### Sociodemographic characteristics of the study participants

A total of 819 mother-child pairs were included in this study with the response rate of 95%. The mean age of mothers/caregivers was (30.3±5.87) years. About 420 (53.7%) children were found within age category of 12–23 months and the rest 362 (46.3%) were infants. There were about 405 (51.8%) female index children (Table 1).

**Table 1:** Sociodemographic and reproductive characteristics of study participants in rural areas of Mareka district, Southern Ethiopia, 2016 (n = 782).

Variables		n	%
Maternal education	No formal education	415	53.1
	Primary	243	31
	Secondary and above	124	15.9
Mothers ethnicity	Dawro	744	95.1
	Others^a^	38	4.9
Religion of mothers	Protestant	723	92.5
	Others^b^	59	7.5
Occupation of mothers	Housewives	747	95.5
	Others^c^	35	4.5
Marital status of mothers	Currently Married	773	98.8
	Previously married ^d^	9	1.2
Paternal education	No formal education	358	45.8
	Primary	289	37
	Secondary and above	135	17.2
Place of delivery	Home	563	72
	Health institution	219	28
Household family size	Mean ± SD	(5.06±1.35)	
	<4	59	7.5
	4–5	463	59.2
	>5	260	33.3
Birth order	Mean ± SD	(2.06±0.95)	
	1	221	28.3
	2–3	482	61.6
	>3	79	10.1

^a^Wolaita/Konta/Amhara

^b^Cattholic/Orthodox

^c^Trader/Student/employee

^d^Divorced/widowed.

### Utilization of growth monitoring and promotion services

The proportion of children who utilized GMP services in the study area found to be 16.9% (95% CI: 14.2–19.6). More than half of the study participants 433 (55.4%) reported that the GMP services were given regularly. However, majority 625 (80.9%) of them did not know about the growth monitoring and promotion chart despite 415(53.1%) of them had family health card during survey (Table 2).

**Table 2:** Community conversation for mothers/caregivers with children 0–23 months in rural areas of Mareka district, Southern Ethiopia, 2016 (n = 782).

Variables		n	%
Community conversation on utilization of GMP services	Yes	213	27.2
	No	447	57.2
	I do not know	122	15.6
Participation of mothers in community conversation	Yes	97	45.5
	No	116	54.5
Community conversation frequency on GMP	Regular	32	33
	Irregular	65	67
Nutrition and health learning materials during community conversation of GMP	Family health card	80	82.5
	Nutrition and healthy leaflets	62	63.9
	Not utilized	13	2

### Reasons for missing and sources of messages for GMP services utilization

**Fig 1:**
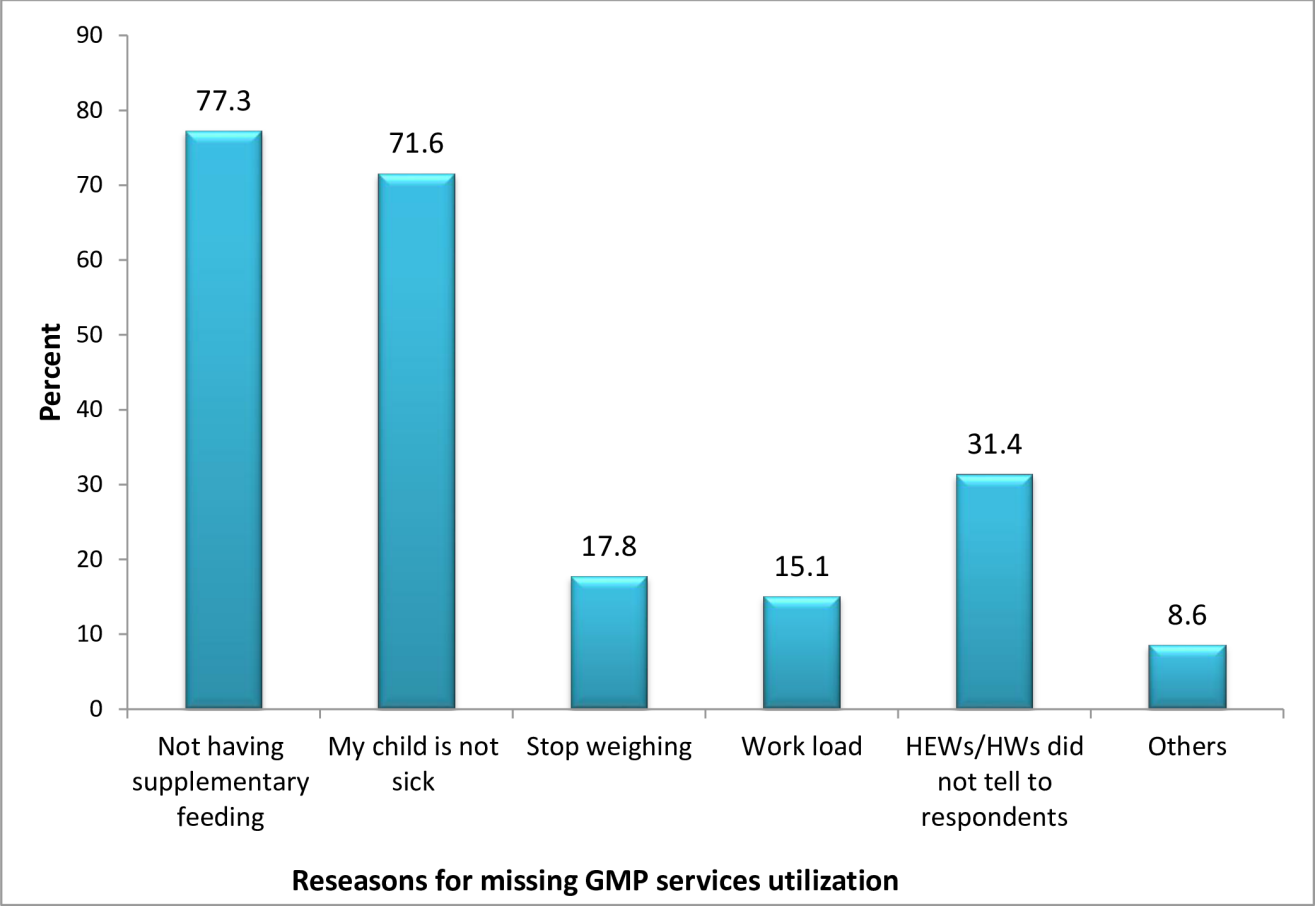
Reasons for missing utilization of GMP sessions in rural area of Mareka District, Southern Ethiopia, 2016 (n = 405).

**Fig 2:**
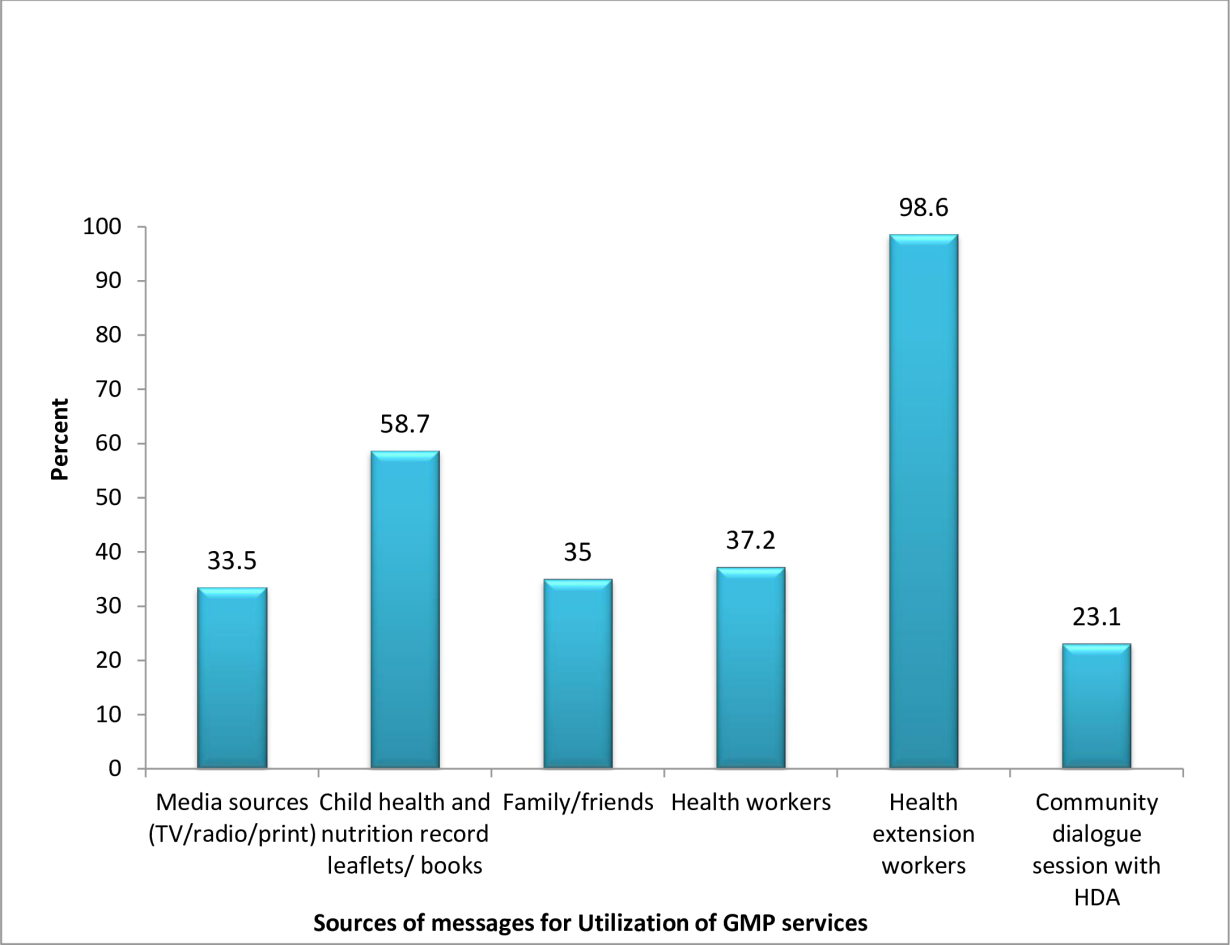
Main source of messages for utilization of GMP services in rural area of Mareka District, Southern Ethiopia, 2016 (n = 782).

The main reasons given by study participants for missing of the GMP sessions were absence of supplementary feeding program 313 (77.3%) and around 290 (71.6%) reported that the child was not sick to attend sessions. Health extension workers did not tell the exact time of GMP session to mothers/caregivers, workloads of mothers/caregivers and child ages not reached to be weighed were also mentioned as reasons (Fig 1) Moreover almost all respondents reported health extension workers771 (98.6%) and community dialogues with health development army team leaders 192 (23.1%) were main source of message for utilization of GMP services (Fig 2).

### Factors associated with utilization of GMP services

The variables such as paternal age, maternal age, paternal education, maternal education, family health card utilization, antenatal care utilization, counselling and postnatal care utilization were associated with the dependent variable in the bivariate regression analysis but they failed to maintain their association with the dependent variable in the multivariable logistic regression analysis. Utilization of GMP services had no significant association with workloads of mother, mothers’/caregivers’ knowledge about growth monitoring and promotion chart, birth order, household latrine and growth monitoring and promotion continuation after full immunization. The multivariable analysis identified young index child’s age, institutional place of delivery, medium and high class wealth index, large family size and regular growth monitoring and promotion frequency as associated factors for utilization of growth monitoring and promotion services (Table 3).

**Table 3:** Predictors of GMP services utilization among children 0–23 months of age in rural communities of Mareka district, Southern Ethiopia, 2016 (n = 782).

Variables		GMP utilization		95% CI		P—Value
		Yes	No	COR	AOR	
Maternal age	<30 years	53	314	1	1	
	≥30 years	79	336	1.39(0.95–2.04)	1.26(0.67–2.38)	0.472
Paternal age	<30 years	23	173	1	1	
	≥30 years	109	477	1.72(1.06–2.78)	1.71(0.83–3.54)	0.147
Counselling	Yes	70	203	1	1	
	No	62	477	2.49(1.70–3.64)	1.52(0.95–2.44)	0.082
GMP frequency	Regular	113	320	6.13(3.68–10.21)	4.37(2.45–7.80)**	0.001
	Irregular	19	330	1	1	
FHC utilization	Yes	90	325	2.14(1.44–3.19)	1.48(0.89–2.45)	0.133
	No	42	325	1	1	
Utilization of ANC services	Yes	90	332	2.05(1.38–3.05)	1.38(0.82–2.31)	0.225
	No	42	318	1	1	
Delivery place	Home	59	504	1	1	
	Health inst	73	146	4.27(2.89–6.31)	3.01(1.65–5.50) **	0.001
Utilization of PNC services	Yes	87	272	2.69(1.8–3.98)	0.97(0.52–1.81)	0.917
	No	45	378	1	1	
Index children age	0–11 months	27	335	1	1	
	12–17 months	48	147	4.05(2.43–6.75)	4.03(2.16–7.51)**	<0.0001
	18-23months	57	168	4.20(2.57–6.90)	3.08(1.70–5.57)**	<0.0001
Family size	<4	21	38	1	1	
	4–5	51	412	0.22(0.12–0.41)	0.14(0.06–0.33)**	<0.0001
	>5	60	200	0.54(0.30–1.00)	0.34(0.14–0.82) *	0.017
Maternal education	No formal	37	378	1	1	
	Primary	54	189	2.92(1.86–4.59)	1.82(0.99–3.51)	0.050
	≥Secondary	41	83	5.05(3.05–8.35)	1.78(0.87–3.63)	0.112
Paternal education	No formal	34	324	1	1	
	Primary	63	226	2.66(1.69–4.17)	1.42(0.74–2.71)	0.291
	≥Secondary	35	100	3.34(1.98–5.62)	1.30(0.61–2.77)	0.495
Wealth index	Low	13	245	1	1	
	Medium	51	212	4,53(2.40–8.57)	3.14(1.51–6.52) ***	0.002
	High	68	193	6.64(3.56–12.38)	3.24(1.59–6.62) ****	0.001

*P_ value < 0.017

***P_value < 0.002

****P_value <0.001

**P_value < 0.001, COR: Crude odds ratio, AOR: Adjusted odds ratio by using binary and multivariable logistic regression.

## Discussion

The overall utilization of GMP services in this study was 16.9%. The present prevalence of GMP services utilization is lower as compared with other studies conducted in Kwazulu Natal (67%) [6]; Uganda (59%), Honduras (87%), Brazil (42%) and Dominican Republic (85%) [4]. This difference might be due to differences in operational definitions, study design, time, and poor understanding and lack of mothers’ participation. This also supported by a qualitative study [8] and an institution based prospective study [9].

In this study mothers/caregivers showed willingness for planned regular attendance of GMP services utilization which is similar with that of 87% reported from Afghanistan [10]. Moreover the majority of participants mentioned the importance of monthly weighing of children. The comparable finding was reported from Ghana [11], where 98.1% of mothers believed the importance of monthly weighing of children.

This study found poor knowledge of mothers/caregivers on GMP chart suggesting that the health professionals’ focus weighing and identifying children’s nutritional status instead of discussing with mothers and communities as reported by [10]. This might be due to heavy workload of health extension workers and low motivations as well as shortage of GMP service tools at health posts.

From multivariable analysis, young index child’s age, delivery in health institution, medium and high class wealth index, large family size and GMP frequency were significantly associated with utilization of GMP services. Women who delivered in health institution were 3.01 times more likely to utilize the GMP services as compared to home delivery. This finding is in agreement with studies reported delivery in health institution as predictors for infant and young child feeding practices, minimum dietary diversity and meal frequency from Northern Ethiopia [12, 13, and 14]. Husbands’ support and participation in budgeting might strengthen this idea reported from India [15]. This might be also due to counselling during antenatal care services [16].

In this study, index child in the age group of 12–23 months found that more likely to utilize the GMP services as compared to infants. This might be due to mothers’ expectation of supplementary foods and fathers’ good knowledge and practices about child health care as supported by one study from Northern Ethiopia [5].

This study found an inverse relationship between family sizes and utilization of GMP services. It might be due to workload of mothers/caregivers at home. This is supported by a report from India showed as a constraint to good child health care [15], not attending antenatal care services, poor socio-economic status and negative attitude of mother’s toward GMP services [13].

Regular attendant mothers/caregivers/ had 4.37 times more likely to utilize GMP services as compared to irregular one. It might be due to the fact that mothers’/caregivers’ at younger age were more likely to be involved in institutional delivery services utilization [12]; leisure time [15]. Media exposure and literacy of mothers reported from different topics related with GMP services may be possible reasons for regular attendants’ more utilization of GMP services [12, 13], influence of father’s good knowledge and practices [5] and influence of community promoters [17]. For irregular attendants, it might be also due to absence of supplementary feeding [5] and presence of sick child [8, 10].

Recall and social desirability bias might be possible limitation for this study. The large sample size and being the first quantitative study in Ethiopia were some of the strengths of this study.

In conclusion, although several efforts have been exerted to promote child growth both at the health institutions and grass root level, the results of GMP services was below the ideal as compared with other countries. Associated factors with utilization of GMP services were index children’s age, institutional place of delivery, wealth index, regular GMP attendance and large family size. The district Health Office can improve the utilization of GMP services through mobilizing health workers and community health development army team leaders. It can also strengthening promotion of institutional delivery, different family planning methods and regular frequency of GMP services particularly for infants through health extension workers and all concerned bodies. Further studies through qualitative method may explore mothers’ perspective to address additional associated factors to low utilization of GMP services.
